# Knowledge, Attitude, and Practice Regarding Diabetic Retinopathy Screening and Eye Management Among Diabetics in Saudi Arabia

**DOI:** 10.7759/cureus.46190

**Published:** 2023-09-29

**Authors:** Taif F Alqahtani, Rahaf Alqarehi, Oyoon M Mulla, Asayel T Alruwais, Shajn S Alsaadi, Hajar Algarni, Yaser M Elhams, Safa Alkalash

**Affiliations:** 1 College of Medicine, Umm Al-Qura University, Makkah, SAU; 2 Ophthalmology, Umm Al-Qura University, Makkah, SAU; 3 Family Medicine, Menoufia University, Shibin el Kom, EGY; 4 Community and Primary Care, Umm Al-Qura University, Al-Qunfudah, SAU

**Keywords:** practice, attitude, knowledge, diabetic retinopathy, diabetes mellitus

## Abstract

Background

Globally, one of the leading causes of blindness is diabetic retinopathy (DR). However, many patients do not participate in DR screening because of a lack of awareness. This study aims to assess the knowledge, attitude, and practice (KAP) level regarding DR screening and eye management among diabetic patients in Saudi Arabia.

Methodology

This cross-sectional study was conducted among diabetic patients aged 18 years or older in Saudi Arabia between October 2022 and February 2023. A validated online KAP-36 questionnaire collected information on sociodemographic data, diabetes profile, diabetes-related complications, and KAP regarding DR screening and management.

Results

Of the 1,391 diabetic patients, 736 (52.9%) had good knowledge about DR screening and care, while 655 (47.1%) had poor knowledge. A positive attitude toward eye examination for the early detection of DR was noticed among 1,124 (80.8%) participants. Regarding the participants’ practice of regular fundus examination, 1,000 (71.9%) participants had good practice. Significant relationships were found between education level (p = 0.017), diabetes mellitus (DM) type and duration (p= 0.01, 0.02), type of treatment (p = 0.001), and a high degree of knowledge. Significant determinants of patients’ favorable attitudes included their type of diabetes (p = 0.003), region of residence (p = 0.038), and work or education outside the medical field (p = 0.001). Age (p = 0.001), location of residence (p = 0.015), educational attainment (p= 0.041), and type of diabetes (p = 0.045) were the factors that determined good practice.

Conclusions

Many diabetic patients supported DR screening and engaged in it regularly. Unfortunately, only around half of the participants had a good understanding of DR. Type 2 diabetes mellitus (T2DM), a longer history of DM, and being highly educated were factors associated with a diabetic patient’s higher level of knowledge. Positive attitudes were significantly higher among those living in the central Saudi region, employed outside of the medical field, and those with T2DM. Finally, regarding the practice of eye screening and management among diabetic patients, elderly patients living in the southern Saudi region and those with T2DM were adherent to their regular eye examinations. Consequently, the key to ensuring adequate adherence to DR screening may be intervention techniques and focused education to increase patients’ knowledge of DR.

## Introduction

Long-term high blood glucose levels mainly cause diabetic retinopathy (DR). Generally, it occurs in patients with type 1, type 2, or gestational diabetes. DR is recognized by retinal ischemia and increased retinal vascular permeability. Prolonged periods of hyperglycemia can damage small blood vessels in the retina, causing hemorrhage, exudates, and retinal swelling. Over time, the retina becomes oxygen-starved, abnormal blood vessels grow incorrectly, and retinal blood vessels leak [1,2]. It is important to note that patients with uncontrolled diabetes can develop other serious complications such as diabetic nephropathy, neuropathy, and cardiomyopathy [3].

DR is by far the most common type of diabetic eye disease. However, other eye diseases can develop, including diabetic macular edema, neovascular glaucoma, and retinal detachment [4,5]. The early stages of DR can occur without any initial symptoms or pain. Nevertheless, a few symptoms can appear as the disease worsens, such as sudden vision changes, blurred vision, eye floaters, spots, double vision, and eye pain [1]. In Saudi Arabia, the diabetes mellitus (DM) prevalence rate in adults is 17.7%, whereas the prevalence of retinopathy ranges between 28.1% and 45.7% [6]. DR accounts for approximately 4.8%-17.5% of vision-threatening conditions among diabetics, with most cases occurring in people aged 50 and older [6,7]. As of 2020, 103 million adults worldwide have DR, which is predicted to increase to 160 million by 2045 [4,8]. Untreated DR not only causes blindness, which is a personal disaster for the individual, but it also raises the community’s economic burden of health care services [9]. Most causes of the DR burden may be due to absenteeism, lost productivity from disease-related absenteeism, unemployment from disease-related disability, and lost productivity due to visual loss from the disease.

DR screening is critical in identifying cases that should be examined and treated without delay to prevent permanent vision loss [10]. Moreover, it serves as the first step toward reducing the problem [11]. Diabetic patients ought to regulate serum cholesterol, blood pressure, and glucose control to reduce DR risk or slow the advancement of the disease [12]. Adults with type 1 DM should have an initial comprehensive eye examination by an ophthalmologist within five years after the onset of the disease, and those with type 2 DM (T2DM) must be screened at the time of diagnosis. If there is evidence of retinopathy, this screening should be done annually or more regularly; otherwise, it should be done every three years [12]. Diabetic women who are pregnant or plan to get pregnant should receive advice on the risk of DR and should have eye exams every trimester and then one year after giving birth, depending on the severity of the DR. The primary care doctor should be informed of the findings of the eye exams [12,13]. Every time a diabetic patient comes in, the primary care physician should inquire about any changes in vision, blurriness, pain, or redness in the eye, and consult an ophthalmologist if any of these symptoms are present. A referral of the patient to an ophthalmologist should be done if it has been over a year since the last eye exam [12].

According to several studies [14-16], many patients do not participate in DR screening because of a lack of awareness. In fact, patients’ awareness of DR was either inadequate or nonexistent [17]. A study was conducted in Riyadh and comprised 404 adult diabetic patients attending outpatient clinics in four hospitals. It revealed that 51% of the patients had poor knowledge of DR screening. In addition, DR was reported by 20% of participants. More than one-fifth of participants were never screened for DR [17]. Hussain et al. concluded that 75% of their participants agreed that patients with DM should undergo regular retinopathy checkups. Still, only 9.6% had undergone eye checkups, and only 9.8% had a follow-up [18]. A study done in Oman revealed excellent knowledge among 72.9% of the study population regarding the diagnosis of DR. Excellent grades of attitude and practice were observed regarding eye involvement and eye check-ups in 18% and 52%, respectively [19]. In Norway, among patients who had been diagnosed with DM, 62.8% had never had an eye exam, and 68.8% had not had an eye exam in more than two years [20]. In Papua New Guinea, a significant fraction of people with DM are still unscreened for DR [16].

Patients’ knowledge and attitudes affect the annual DR screening, primary diabetes prevention, and adherence to the recommendations related to eye care. Therefore, this study aims to assess the knowledge, attitude, and practice (KAP) regarding DR screening among diabetic individuals in Saudi Arabia with a view to identifying gaps in awareness, informing future educational initiatives, and thereby improving health outcomes.

## Materials and methods

Study design and population

This cross-sectional study was conducted in Saudi Arabia between October 2022 and January 2023. This study aimed to assess KAP regarding DR screening and eye management among diabetic patients in Saudi Arabia. The inclusion criteria were Saudi and non-Saudi diabetic patients aged 18 years and older who consented to participate in the survey. The exclusion criteria were any non-diabetic person below the age of 18 years and living outside Saudi Arabia.

Study setting

The Kingdom of Saudi Arabia is one of the largest Arab countries in the Arabian Peninsula covering about 80% of the region. Geographically, Saudi Arabia includes the following six regions: Eastern, Central, Northern, Northwest, Midwest, and Southwest; these regions comprise 13 provinces. These provinces are further subdivided into 118 governorates. The population size, including expats in 2023, amounts to 36.33 million. Expats make up more than a third of Saudi Arabia’s population. In 2021, the expat population was 13.49 million [21].

Sampling procedure

The sample size was calculated using Open Epi Version 3.0 (www.openepi.com), given that the estimated number of diabetic patients in Saudi Arabia is around seven million [19]. The sample size calculation was based on a 95% confidence interval (CI) with a 50% frequency percentage. A sample of 384 diabetic patients was required; we intended to maximize the sample size to reach 1,391 participants. The sample was distributed equally among the five regions of Saudi Arabia.

Data collection tool

An organized, self-administered, online questionnaire was used to collect data. The questionnaire was modified and adopted from another survey study [22]. An Arabic version was used to make it suitable for patients to understand. The questionnaire contained the following five sections: the participant’s demographic data and the disease characteristics, questions regarding the individual’s diabetic complications, the individual’s knowledge and understanding of DR screening and eye management, questions about individuals’ attitudes regarding the screening and management of the eye in diabetes, and the last part related to the individuals’ practice and lifestyle as a person with diabetes. The questions on the complications, knowledge, and practice sections were multiple choice (yes and no responses). At the same time, attitude responses were based on a five-point Likert scale. The sum of knowledge and attitude questions was graded as *good *and *positive*, respectively, if the score was 60% or higher. The practice was considered *safe *if the sum of all the responses in this section was greater than 50%; anything below this level was regarded as *unsafe* practice toward DRS and eye management. A pilot study was conducted among 40 patients to avoid interobserver variation or bias. To determine the reliability of the questionnaire, Cronbach’s alpha coefficient was calculated as 0.77 for the knowledge part, 0.83 for the practice part, and 0.69 for the attitude part.

Data collection procedure

The primary research data was collected using a predesigned survey circulated on several electronic platforms such as WhatsApp, Twitter, Snapchat, and Facebook over four months from October 2022 to January 2023. The survey included a mandatory question concerning whether the respondent was a diabetic patient and lived in Saudi Arabia, and anyone who did not have diabetes and was from outside Saudi Arabia could not continue filling out the survey. The total number of questionnaires completed, as determined by analyzing the data assembled, was 1,391. The study sample was obtained. Because the questionnaire was created with the proper response format, there were no unanswered questions.

Statistical analysis

Descriptive analysis was performed based on the frequencies and percentages calculated for categorical variables. A chi-square test was used to assess the associations between the variables. Nonparametric, univariate analysis was performed as an alternative for data that did not assume normal distributions. A Pearson correlation coefficient was computed to assess the linear relationship between knowledge and attitude, knowledge and practice, and attitude and practice. All tests were judged to be significant at p-values of 0.05. The data were analyzed using SPSS software for Mac, version 26 (IBM Corp., Armonk, NY, USA).

Ethics approval

Informed consent was obtained from the participants before filling out the questionnaire. The study was performed after obtaining approval from the Umm Al-Qura University Biomedical Research Ethics Committee of Umm Al-Qura University (approval number: APO-02-K-012-2022-11-1228).

## Results

A total of 1,391 diabetic patients fulfilling the inclusion criteria completed the questionnaire. Among the participants, 859 (61.8%) were female. Most of them, 565 (40.6%), were between 18 and 30 years of age, and 258 (18.5%) were between 41 and 50 years of age. As for educational level, 816 (58.7%) patients had a university degree, and 168 (12.1%) had a low level of education. Most participants had T2DM (652, or 46.9%), while 350 (25.2%) did not know what type they had. The demographics and diabetic profile of the participants are given in Table 1.

**Table 1:** Demographic and diabetes profile of the diabetic patients. T1DM: type 1 diabetes mellitus; T2DM: type 2 diabetes mellitus; DM: diabetes mellitus

Personal data	N	%
Gender		
Male	532	38.2%
Female	859	61.8%
Age in years		
18–30	565	40.6%
31–40	175	12.6%
41–50	258	18.5%
51–60	242	17.4%
>60	151	10.9%
Region of Saudi Arabia		
Central	178	12.8%
Southern	266	19.1%
Western	376	27%
Northern	259	18.6%
Eastern	321	22.4%
Educational level		
Below high school	168	12.1%
High school	247	17.8%
Diploma	160	11.5%
College degree	741	53.3%
Higher degree	75	5.4%
Working/studying in the health field		
Yes	316	22.7%
No	1,075	77.3%
Type of DM		
T1DM	335	24.1%
T2DM	652	46.9%
Other	54	3.9%
I don’t know	350	25.2%
Duration of DM		
Less than 1 year	325	23.4%
1-5 years	331	23.8%
Over 5 years to 10 years	260	18.7%
Over 10 years to 15 years	244	17.5%
Over 15 years	231	16.6%
Treatment for DM		
Diet	213	15.3%
Medication	573	41.2%
Injection	429	30.8%
None	176	12.7%

**Figure 1:**
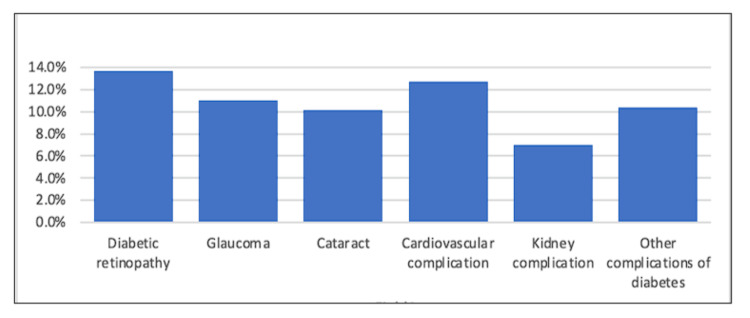
Complications of diabetes mellitus among diabetic patients.

In terms of the complications of DM that diabetic patients self-reported, 991 (71.2%) participants did not report any, while DR was the most common eye complication among 191 (13.7%) diabetic patients. Glaucoma and cataracts were then found in 161 (11.6%) and 139 (10.0%), respectively (Figure 1).

**Figure 2:**
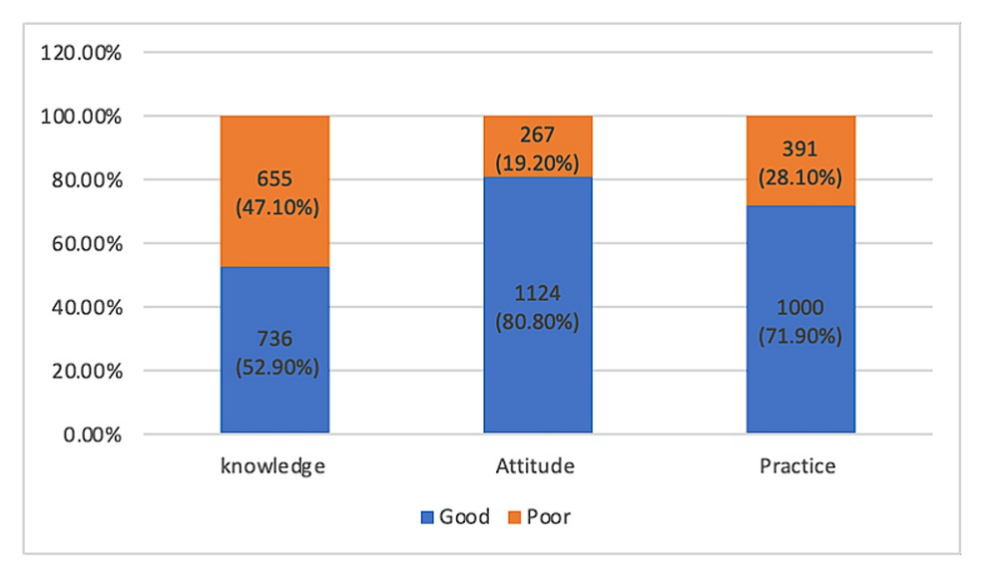
The knowledge, attitude, and practice regarding diabetic retinopathy screening and management among participants.

The KAP regarding DR screening and management among participants revealed a good level of knowledge of the eye complications of diabetes, and its management was noted in 736 (52.9%) patients. A positive attitude toward regular eye examination and screening for DR was noted in 1,124 (80.8%) of the participants. Good practice of fundus examination by the participants was predominant in 1,000 (71.9%) participants (Figure 2).

The determinants of knowledge regarding DR screening and management among diabetic patients were examined and revealed that a good level of knowledge (the score was 60% or higher) was significantly associated with a higher educational level (p = 0.017). A higher level of knowledge was noted in T2DM (59.4%) than in type 1 DM (T1DM) (54.3%), which also indicates a significant association (p = 0.01). Other determinants that were found to be of marked value include duration since DM diagnosis (p = 0.02) and type of treatment (p = 0.001). Likewise, patients who used injections as a mode of therapy manifested the highest percentage of knowledge (61.8%) (Table 2).

**Table 2:** Factors associated with diabetic patients’ knowledge regarding diabetic retinopathy screening. A p-value less than 0.05 is significant. T1DM: type 1 diabetes mellitus; T2DM: type 2 diabetes mellitus; DM: diabetes mellitus; P: Pearson chi-square test

Factors		Overall knowledge level				P-value
		Poor		Good		
		No	%	No	%	
Region of Saudi Arabia	Western region	98	55.1%	80	44.9%	0.107
	Central region	133	50.0%	133	50.0%	
	Northern region	171	45.5%	205	54.5%	
	Eastern region	115	44.4%	144	55.6%	
	Southern region	138	44.2%	174	55.8%	
Age in years	18–30	285	50.4%	280	49.6%	0.291
	31–40	83	47.4%	92	52.6%	
	41–50	113	43.8%	145	56.2%	
	51–60	108	44.6%	134	55.4%	
	>60	66	43.7%	85	56.3%	
Gender	Male	251	47.2%	281	52.8%	0.957
	Female	404	47.0%	455	53.0%	
Educational level	Below high school	78	46.4%	90	53.6%	0.017*
	High school	136	55.1%	111	44.9%	
	Diploma	83	51.9%	77	48.1%	
	College degree	329	44.4%	412	55.6%	
	Higher degree	29	38.7%	46	61.3%	
Working/studying in the health field	Yes	148	46.8%	168	53.2%	0.918
	No	507	47.2%	568	52.8%	
Type of DM	T1DM	153	45.7%	182	54.3%	0.001*
	T2DM	265	40.6%	387	59.4%	
	Other	27	50.0%	27	50.0%	
	I don’t know	210	60.0%	140	40.0%	
Duration of DM	Less than 1 year	174	53.5%	151	46.5%	0.023*
	1–5 years	147	44.4%	184	55.6%	
	Over 5 years to 10 years	118	45.4%	142	54.6%	
	Over 10 years to 15 years	122	50.0%	122	50.0%	
	Over 15 years	94	40.7%	137	59.3%	
Treatment for DM	Diet	121	56.8%	92	43.2%	0.001*
	Medication	261	45.5%	312	54.5%	
	Injection	164	38.2%	265	61.8%	
	None	109	61.9%	67	38.1%	

The factors associated with diabetic patients’ attitudes toward DR screening and management are presented. It was found that the region of residence influenced patients’ attitudes (p = 0.038), with the central region being the best in this regard (85.0%), in contrast to the southern region (75.6%). Strikingly, patients working or studying in the health field were found to embrace a less positive attitude toward DR screening compared with other patients (only 73.7% in contrast to 82.9%). Like the knowledge level, a significant association with the type of diabetes was noted (p = 0.03), in which T2DM patients showed a higher percentage (84.2%) compared to T1DM (79.4%) (Table 3).

**Table 3:** Factors associated with diabetic patients’ attitudes regarding diabetic retinopathy and management A p-value less than 0.05 is significant. T1DM: type 1 diabetes mellitus; T2DM: type 2 diabetes mellitus; DM: diabetes mellitus; P: Pearson chi-square test

Factors		Attitude level				P-value
		Negative		Positive		
		No	%	No	%	
Region of Saudi Arabia	Western region	39	21.9%	139	78.1%	0.038*
	Central region	40	15.0%	226	85.0%	
	Northern region	66	17.6%	310	82.4%	
	Eastern region	46	17.8%	213	82.2%	
	Southern region	76	24.4%	236	75.6%	
Age in years	18–30	119	21.1%	446	78.9%	0.337
	31–40	33	18.9%	142	81.1%	
	41–50	53	20.5%	205	79.5%	
	51–60	38	15.7%	204	84.3%	
	>60	24	15.9%	127	84.1%	
Gender	Male	95	17.9%	437	82.1%	0.319
	Female	172	20.0%	687	80.0%	
Educational level	Below high school	28	16.7%	140	83.3%	0.753
	High school	53	21.5%	194	78.5%	
	Diploma	33	20.6%	127	79.4%	
	College degree	138	18.6%	603	81.4%	
	Higher degree	15	20.0%	60	80.0%	
Working/studying in the health field	Yes	83	26.3%	233	73.7%	0.001*
	No	184	17.1%	891	82.9%	
Type of DM	T1DM	69	20.6%	266	79.4%	0.003*
	T2DM	103	15.8%	549	84.2%	
	Other	18	33.3%	36	66.7%	
	I don’t know	77	22.0%	273	78.0%	
Duration of DM	Less than 1 year	78	24.0%	247	76.0%	0.057
	1–5 years	57	17.2%	274	82.8%	
	Over 5 to 10 years	55	21.2%	205	78.8%	
	Over 10 to 15 years	38	15.6%	206	84.4%	
	Over 15 years	39	16.9%	192	83.1%	
Treatment for DM	Diet	46	21.6%	167	78.4%	0.153
	Medication	103	18.0%	470	82.0%	
	Injection	75	17.5%	354	82.5%	
	None	43	24.4%	133	75.6%	

The responses to practices related to DR screening and management by people with diabetes were associated with the determinants. A good level of practice was positively associated with the region of residence (p = 0.015); in contrast to what was observed in the level of knowledge, the southern region recorded the safest practice (35.6%). Additionally, older age was associated with safer practice, as 41.7% of those aged 60 years and older showed better DR screening practices compared to 23.2% of younger patients (18-30 years old) (p = 0.001). An exceptional finding of a reverse relation of education level to practice was noted, where 38.1% of those with education below high school practiced DR screening better than those holding a college degree (p = 0.041). The following factors were found to be significant concerning the patients’ safe practice: T2DM, a longer duration since diagnosis, and treatment with injections (p = 0.001) (Table 4).

**Table 4:** Factors associated with diabetic patients’ practice toward diabetic retinopathy and management. A p-value less than 0.05 is significant. T1DM: type 1 diabetes mellitus; T2DM: type 2 diabetes mellitus; DM: diabetes mellitus; P: Pearson chi-square test

Factors		Practice level				P-value
		Poor		Good		
		No	%	No	%	
Region of Saudi Arabia	Western region	129	72.5%	49	27.5%	0.015*
	Central region	196	73.7%	70	26.3%	
	Northern region	286	76.1%	90	23.9%	
	Eastern region	188	72.6%	71	27.4%	
	Southern region	201	64.4%	111	35.6%	
Age in years	18–30	434	76.8%	131	23.2%	0.001*
	31–40	129	73.7%	46	26.3%	
	41–50	186	72.1%	72	27.9%	
	51–60	163	67.4%	79	32.6%	
	>60	88	58.3%	63	41.7%	
Gender	Male	372	69.9%	160	30.1%	0.199
	Female	628	73.1%	231	26.9%	
Educational level	Below high school	104	61.9%	64	38.1%	0.041*
	High school	183	74.1%	64	25.9%	
	Diploma	115	71.9%	45	28.1%	
	College degree	541	73.0%	200	27.0%	
	Higher degree	57	76.0%	18	24.0%	
Working/studying in the health field	Yes	230	72.8%	86	27.2%	0.688
	No	770	71.6%	305	28.4%	
Type of DM	T1DM	395	74.7%	134	25.3%	0.001*
	T2DM	238	71.0%	97	29.0%	
	Other	434	66.6%	218	33.4%	
	I don’t know	40	74.1%	14	25.9%	
Duration of DM	Less than a year	288	82.3%	62	17.7%	
	1–5 years	271	83.4%	54	16.6%	0.001*
	Over 5 years to 10 years	271	81.9%	60	18.1%	
	Over 10 years to 15 years	187	71.9%	73	28.1%	
	Over 15 years	145	59.4%	99	40.6%	
Treatment for DM	Diet	126	54.5%	105	45.5%	
	Medication	162	76.1%	51	23.9%	0.001*
	Injection	411	71.7%	162	28.3%	
	None	265	61.8%	164	38.2%	

The correlation between knowledge, attitude, and practice was evaluated using Pearson’s correlation analysis and interpreted using the following criteria: 0-0.25 = weak correlation, 0.25-0.5 = fair correlation, 0.5-0.75 = good correlation, and greater than 0.75 = excellent correlation. It revealed that there was a significant positive correlation between knowledge and attitude (r = 0.14, p = 0.001), knowledge and practice (r = 0.27, p = 0.001), and, finally, a positive correlation between attitude and practice was detected (r = 0.8, p = 0.008) (Table 5).

**Table 5:** Correlation analysis between the study patient’s knowledge, attitude, and practice regarding diabetic retinopathy and screening. A p-value less than 0.05 is significant.

Variable	Correlation coefficient	P-value
Knowledge-Attitude	0.14	0.001
Knowledge-Practice	0.27	0.001
Attitude-Practice	0.8	0.008

## Discussion

In this self-reported, cross-sectional study, we aimed to assess the level of KAP regarding DR screening and eye disease management among diabetic patients in Saudi Arabia. A previous study found that 51% of people with diabetes had little understanding of DR screening. More than one-fifth had never had a DR screening [21]. Consistently, our findings showed that a good level of knowledge of eye disease in diabetes and its management was noted in 736 (52.9%) patients. A positive attitude was noted in 1,124 (80.8%). Good practice was indicated in 1,000 (71.9%). The findings of earlier studies on this topic indicate that additional evaluation of KAP about DR screening and eye management, as well as its associated factors, is necessary to support improving their KAP level [6,8,12]. The variance between the current study’s findings and the previous ones may be related to the differences in the characteristics of the studied populations and sample sizes.

Furthermore, a significant relationship (p = 0.01) was seen between the level of knowledge in T2DM (59.4%) and T1DM (54.3%). Higher education levels were also significantly associated with higher knowledge levels (p = 0.017). Unsurprisingly, educated patients have more opportunities to learn and read about health and disease. Contrary to our result, another study found that the degree of education of diabetes patients was unrelated to their KAP level [22]. Based on the results of the current study, duration since diagnosis (p = 0.02) and type of diabetes therapy (p = 0.001) are other factors identified as having a significant impact on their understanding. The largest proportion of knowledge (61.8%) is shown by patients who received treatment by injection. In contrast to a prior study, the length of diabetes was not connected to the KAP level [22]. According to another study, those who are using insulin will engage with medical personnel more frequently. This accounts for the high proportion of positive attitudes toward health checks, particularly eye exams [19].

The research found that patients’ attitudes were influenced by their location of residence (p = 0.038). Compared to the southern area’s 75.6%, the central region performs the best (85.0%). The central region has more educational and healthcare services, so patients in this area have more opportunities than others. The unexpected finding that those in the health field did not have a more positive attitude toward DR screening is intriguing (p = 0.001). This finding should be searched for its origin through conducting qualitative research and using focus group discussions to deeply understand the perceptions of diabetic patients who are working in healthcare services about DR screening. A significant relationship between the type of diabetes and knowledge level was also found (p = 0.03), with T2DM patients showing a greater proportion (84.2%) than T1DM patients (79.4%). Worth noting is that a study found that lower rates of preventative treatment usage relate to limited health literacy [23]. According to findings from another study, T2DM patients demonstrated low adherence rates to eye care [24].

Our study showed that diabetics’ responses to practice about DR screening and management were linked to the factor of residency. In contrast to what was seen in the degree of knowledge, the southern area registered the safest practice (35.6%). However, research in 2020 in Riyadh [19] found that just 25% of the sample performed yearly DR screening. Compared to a 2016 survey conducted in Al Jouf and Hail Provinces, research revealed that around 95% of people underwent routine eye examinations [25]. This study found that older patients with T2DM for a longer duration possessed better practice of DR screening, which is supported by an American study that revealed that older age, female gender, native language other than English, and attendance at auxiliary diabetes clinic appointments are characteristics that promote the adherence to DR screening [26]. Furthermore, another study reported that the majority of individuals with newly discovered type 2 diabetes did not achieve the standards for DR screening as currently advised [27].

This study showed a significant positive correlation between knowledge and attitude (r = 0.14, p = 0.001) and knowledge and practice (r = 0.27, p = 0.001). Finally, a positive correlation between attitude and practice was detected (r = 0.8, p = 0.008). This was similarly determined in another study conducted in South India, where it showed a significant association with good knowledge (p < 0.001), a positive attitude (p < 0.001), and a good practice culture (p = 0.003) [18]. Neither attitude was significantly associated with any determinants in another study that took place in a private hospital in Riyadh [22].

Limitations

The following are the study’s limitations: first and foremost, the online self-administered questionnaire may have had the disadvantage of only applying to individuals who can read, are familiar with internet technologies, and have internet access. As a result, the study has not assessed the degree of awareness about DR among many people predisposed to it and its effects. Moreover, the virtual collection of data may affect its credibility, especially when we assess the complications of DM among diabetic patients and their practice of regular fundus examination. This drawback could be corrected by using targeted interviews with diabetic patients in any healthcare facility, whether at the primary or secondary care level. Second, the non-probability sampling technique may be susceptible to selection bias. Therefore, we advise employing random sampling approaches to conduct additional research on this subject.

## Conclusions

Most diabetes patients were supportive of DR screening and engaged in it regularly. Unfortunately, only around half of the participants had a good understanding of DR. When patients are unaware of the importance of early detection, interventions from healthcare facilities to increase access to diabetic eye exams may not yield favorable results. Being highly educated, having T2DM, and having a longer history of DM are all characteristics linked to patients’ deeper understanding of DR. Those with T2DM, those working outside the healthcare sector, and those residing in central Saudi Arabia had much more positive attitudes. In terms of practicing DR screening, older patients who resided in the southern region of Saudi Arabia, were low educated, and had T2DM consistently went for eye examinations. Consequently, the key to ensuring adequate adherence to DR screening is focused education. It is recommended that health authorities coordinate with media houses to raise awareness of DR screening, especially in countries like Saudi Arabia, where the epidemic proportion of diabetes and DR is high. Last but not least, additional research in the form of interactive interviews with diabetic patients in family medicine or ophthalmology clinics is highly recommended. This will enable a better understanding of their perspectives on routine fundus examinations for identifying DR in its early stages. Additionally, during these interviews, a review of their medical records should be done to find out whether routine fundus examinations are being performed. Qualitative research through focus group discussions is needed to deeply understand the perceptions of diabetic patients who are working in healthcare services about DR screening, which is highly recommended. We further advocate conducting another study on family physicians’ awareness and practice of DR, as well as the need for regular fundus examinations in diabetes patients.
